# Metformin Use and Risk of All-Cause Mortality and Cardiovascular Events in Patients With Chronic Kidney Disease—A Systematic Review and Meta-Analysis

**DOI:** 10.3389/fendo.2020.559446

**Published:** 2020-10-07

**Authors:** Yao Hu, Min Lei, Guibao Ke, Xin Huang, Xuan Peng, Lihui Zhong, Ping Fu

**Affiliations:** ^1^Department of Medicine Renal Division, West China Hospital, West China School of Medicine, Sichuan University, Chengdu, China; ^2^Department of Medicine Renal Division, Affiliated Hospital & Clinical Medical College of Chengdu University, Chengdu, China; ^3^Department of Medicine Renal Division, West China Hospital, West China School of Medicine, Sichuan University, Kidney Research Institute, Chengdu, China

**Keywords:** metformin and mortality in CKD, cardiovascular events, meta-analysis, type 2 diabetes, chronic kidney disease

## Abstract

**Background:**

To evaluate whether metformin use assuredly alters overall all-cause death in patients with type 2 diabetes mellitus (T2DM) and chronic kidney disease (CKD).

**Methods:**

Pubmed, Web of Science, Embase, and Cochrane Central Register of Controlled Trials were systematically searched from inception to Feb. 29, 2020 with no language restriction. All related articles comparing all-cause death of T2DM and CKD patients after metformin use (monotherapy or combination) versus non-metformin treatment were identified. Pooled risk ratios (RR) and 95% confidence intervals (CI) were computed using random-effects models regardless of the heterogeneity quantified by Cochrane χ^2^ and I^2^ statistics.

**Results:**

Totally 13 studies (9 cohort studies [CSs], 3 subanalyses or post-hoc analyses of randomized controlled trials [RCTs], and 1 nested case-control article) involving 303,540 patients were included. Metformin-based treatments relative to any other measure displayed significantly lower risks of all-cause mortality (Pooled RRs 0.71, 95%CI 0.61 to 0.84; I^2 =^ 79.0%) and cardiovascular events (Pooled RRs 0.76, 95%CI 0.60 to 0.97; I^2 =^ 87.0%) in CKD patients at stage G1-3, with substantial heterogeneity. Metformin use was not significantly related with these end points in advanced CKD patients.

**Conclusions:**

Metformin use is connected with significantly less risks of all-cause mortality and cardiovascular events in patients with T2DM and mild/moderate CKD. However, RCTs with large sample sizes are warranted in the future to assess whether these key benefits extend to later stages of CKD by dose adjustment.

## Introduction

Chronic kidney disease (CKD) is a global public health issue and gradually becomes more prevalent, mainly due to an increment in the diagnosis of type 2 diabetes mellitus (T2DM) (1, 2). CKD attacks about 43% of patients with type 2 diabetes, and its incidence is up to 61% in the diabetic people of US at age 65 years and above (3). Metformin is a biguanide diabetic drug that delays intestinal glucose absorption and glucose generation in the liver and intensifies insulin sensitivity, since not all effects of metformin are clear so far (4). Metformin is commonly used in the first-line for type 2 diabetic patients because of its low cost, effectiveness, weight independence, and cardiovascular benefits (5, 6). However, metformin has been banned from kidney dysfunctional patients because of the suspected risk of lactic acidosis. Pharmacologically, after 3b CKD patients took 1,000 mg/d and G4 CKD patients used 500 mg/d for 4 months, the serial blood metformin levels were always below the upper limit of normal level, and lactate levels were kept at ≤ 5.0 mmol/L (7). Similarly, two recent systematic reviews reported a lack of evidence that metformin relative to other antidiabetic drugs can raise the occurrence of lactic acidosis (8, 9). In another systematic review, metformin intake decreased the all-cause deaths of moderate CKD patients (10), but this review had many limitations, including the lack of subgroup analysis, and ignorance of heterogeneity and publication bias. Evidence now warns that gentle to mild CKD people should use metformin carefully (9, 11).

Nevertheless, metformin is yet prescribed to elderly advanced-CKD patients with DM in Canada (12). It is unknown whether metformin is safe for advanced CKD patients (13, 14), and more recent instructions suggest careful use for these patients until more confirmed evidence about its safety is reported (15). Recent proofs also give rise to conflicting results about metformin use with the presence of both type 2 diabetes and advanced CKD. As reported, supplementation with metformin is probably unlinked to a lower all-cause death rate among advanced CKD patients (15, 16). However, it is also believed that mortality rates will considerably differ after metformin use (17, 18). In particular, some undetected confounders (e.g., study design, renal dysfunction criteria, ignorance of model adjustment, dissimilar comorbidity profile) may result in bias. For these reasons, we planned to explore how metformin will impact the risk of death rates reported among the existing and up-to-date studies.

## Methods

### Search Scheme and Inclusion Criteria

Bibliographical searching on EMBASE, Pubmed, Web of Science, and Wiley Cochrane Central Register of Controlled Trials was finished independently by Y.H. and M.L. (any date up to 29 February, 2020) as per relevant guidance (19). Disagreement was resolved by consensus or by consultation with a third investigator. The study types of interest were clinical trial, observational study (OS), post-hoc analysis or subanalysis of randomized controlled trial (p-h/sa of RCT), and review. No language limitation was applied. The broad key words included “metformin”, “Glucophage”, “dimethylbiguanidine”, “dimethylguanylguanidine”, “dimethylbiguanidium”; “chronic kidney disease”, “CKD”, “chronic renal failure”, “CRF”, “chronic renal dysfunction”, “renal insufficiency”; “dialysis” and “NIDDM”, “type 2 diabet*”, “type II diabet*”, “T2D”, “T2DM”; “death”, “die”, “dead”, “decease”, “mortality”, and “mortalit*”. The search scheme was elaborated in Additional file 1 and the consistency with PRISMA Statement for reporting Systematic Review and Meta-analysis was illustrated in Additional file 2. When detailed information needed for the analysis was unavailable, the original authors will be contacted through e-mail to obtain the missing information.

### Inclusion and Exclusion Criteria

Inclusion criteria were: (I) RCT, p-h/sa of RCTs, or OSs; (II) provision of endpoints for all-cause mortality and cardiovascular events in patients with T2DM and CKD (regardless of CKD stage or dialysis type) with or without metformin use. The tested outcomes were assured by physical tests and hospital records, or recognized from links of administrative records. The exclusion criteria were: (I) kidney transplant; (II) case report, comment, editorial, letter, quasi-experiment (non-random subject assignment), or unpublished study; (III) abstract or conference proceeding. Of two or more articles from the same team or organization, only the one with latest publication or largest sample size was selected.

### Data Isolation and Quality Assessment

Two investigators independently extracted all information of interest in standardized form, including demographic characteristics of patients (age, gender, race/ethnicity), stage of CKD, duration of DM, and follow-up duration. Other data of concern included study information (selection criteria), type, intervention, and clinical outcomes (all-cause death, cardiovascular events), analysis scheme (statistical models, adjusting variables), and effect levels (hazard ratio [HR], risk ratio[RR], odds ratio [OR] or raw data for re-computation). We acquired effect estimates from models with minimal or full adjustment and reporting the adjusting variables.

The quality of each p-h/sa of RCT or OS was assessed by Y.H. and M.L. with a 9-star Newcastle–Ottawa scale (20, 21), and high quality was implied by a score > 6 stars. The evidence quality of each outcome among the enrolled articles was assessed as deficient, low, modest or high by YH and ML independently according to Grading of Recommended Assessment, Development and Evaluation (22). Any inconsistency between them was addressed *via* discussion.

### Statistical Analysis

The first and second end points were all-cause mortality and cardiovascular events, respectively, as per the staging of CKD. Dichotomous outcomes were synthesized using HR, RR or OR with 95% confidence interval (CI). If an article presented more than one result of the relationship between metformin intake and risk of key clinical outcomes as per subtypes (e.g., by weaker kidney function), a within-article overall estimate was acquired. Natural logarithm of RRs (logRRs) and standard errors were pooled by DerSimonian and Laird’s approach in a random-effects model. The 95%CI with null “1” indicated no clinical significance even if P < 0.05.

Given the a-priori dissimilarity of the enrolled studies, stratified analyses by study design (p-h/sa of RCT, OS) was pre-specified if the data were enough. I^2^ ≥50%, 25%–50%, and <25% indicated high, gentle and low inter-study heterogeneity respectively. Any identifiable source of dissimilarity was found out by sensitivity test, in which the articles were removed one by one.

Publication bias was examined by Begg’s and Egger’s tests when 5 or more studies were present for analysis, and by visually testing the dissymmetry of funnel plots of estimated effects versus standard errors (23). Any publication bias (P<0.10) was processed by Duval & Tweedie’s trim-and-fill approach. Other statistical analyses were finished on STATA 12.0 (STATA College Station, USA) at significance level of two-sided *P* < 0.05.

## Results

### Study Selection, Characteristics, and Quality Assessment

**Figure 1:**
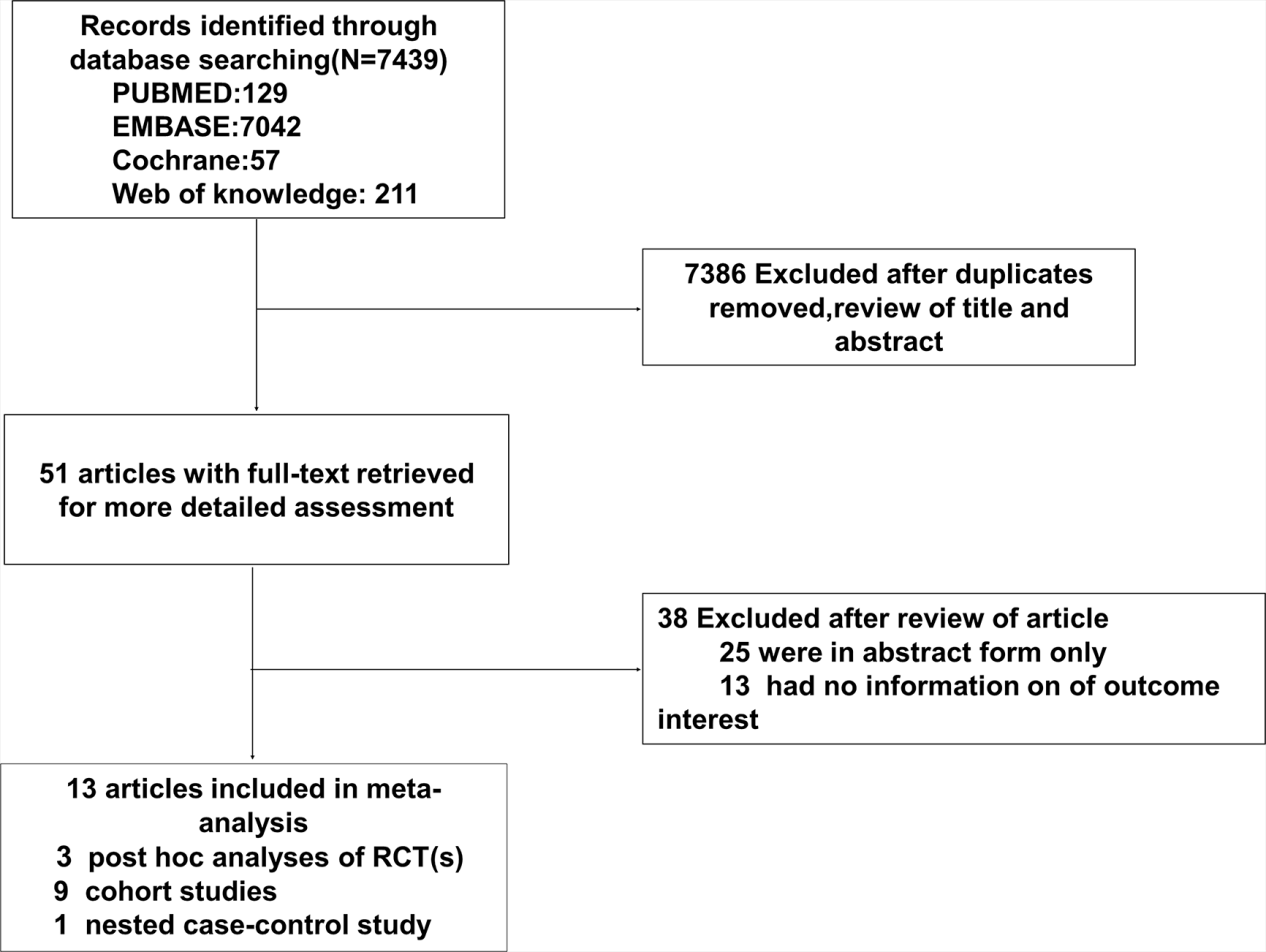
Flow-diagram of study selection.

initial search found 7,439 potentially feasible articles, and after title and abstract screening, 51 articles were retrieved for full-text assessment. Finally, 13 studies were included, including 3 p-h/sa of RCTs (16, 24, 25), 9 cohort studies (CSs) (15, 18, 26–32), and 1 nested case-control study (17) (**Figure 1**). Of the 13 studies, sample sizes varied from 1016 to 175,296, mean age of patients was 55.5 to 69.6 years, female proportion was 1.57% to 59.31%, duration of DM was 5.9 to 14.6 years, and follow-up duration was 1 to 7.3 years. Except for the multinational origins in 3 studies (16, 24, 25), other origins included Europe and America in 7 articles (17, 26–29, 31, 32), and Asia in 3 articles (15, 18, 30). The control was set as sulfonylurea in 3 studies (29, 31, 32), lifestyle modification in 1 article (30), thiazolidinedione in 1 study (26), glyburid and insulin in 1 study (17), and metformin non-users in the rest studies. CKD definitions varied and were eGFR-based in 10 studies and serum creatinine-based in the other three studies (15, 26, 29). All studies adjusted for several baseline participant discrepancies between metformin use and non-use; 7 utilized propensity scores. All studies presented data with all-cause mortality, and four studies reported data of cardiovascular events (25, 28, 30, 32). **Table 1** summarized the major information of the 13 studies.

**Table 1:** Detailed demographic characteristics and outcomes of studies included in the meta-analysis.

Study	Sites	Studydesign	Sample size	Baseline years	Comparison group	Female(%Total)	Follow-up Duration (Years): Metformin users/ Comparison group	Mean Age (Years old)	HbA1c(%)	Duration of Diabetes (years)	BMI (kg/m2)	Smoker(%Total)
Masoudi et al. (26)	The United States	Cohort study	5859	1998-1999 or 2000-2001	Thiazolidinedione	NR	1/1	NR	NR	NR	NR	NR
Roussel et al. (16)	Multinational	Post hoc analysis of RCT	16,535	2003-2004	Metformin Non-users	NR	1.7/1.7	NR	NR	NR	NR	NR
Aguilar et al. (27)	The United States	Cohort study	1246	2000-2002	Metformin Non-users	NR	2/2	NR	NR	NR	NR	NR
Weir et al. (17)	Canada	Nested case-control	1644	1997-2008	Glyburid Insulin	51.3	NR	NR	NR	NR	NR	NR
Ekström et al. (28)	Sweden	Cohort study	51,675	2004-2010	Metformin Non-users	41.9	3.9/3.9	65.3	7.3	9.4	29.5	14.0
Morgan et al. (29)	The United Kingdom	Cohort study	11,481	2000-2012	Sulphonylurea	NR	2.9/3.1	NR	NR	NR	NR	NR
Fung et al. (30)	Hong Kong	Cohort study	6800	2008	Lifestyle modifications.	59.31	5.2/3.6-3.7	62.57	6.57	NR	25.59	5.96
Hung et al. (15)	Taiwan	Cohort study	3252	2000-2009	Metformin Non-users	51.0	2.1/2.1	67.2	NR	5.9	NR	NR
Marcum et al. (31)	The United States	Cohort study	175,296	2004-2009	Sulfonylurea	1.57	1.7/1.7	65.44	NR	NR	NR	NR
Bergmark et al. (24)	Multinational	Post hoc analysis of RCTs	1332	NR	Metformin Non-users	43.17	2.1/2.1	69.6	7.62	14.6	31.33	8.93
Charytan et al. (25)	Multinational	Post hoc analysis of RCT	1016	2004-2009	Metformin Non-users	35.83	2.4/2.4	67.5	6.85	14.1	31.4	3.4
Kwon et al. (18)	Korea	Cohort study	5408	2001-2016	Metformin Non-users	42.45	7.3/7.3	67.4	7.4	NR	18.95	NR
Whitlock et al. (32)	Canada	Cohort study	21,996	2006-2017	Sulfonylurea	48.8	1.4/1.1	55.5		NR	NR	NR

Study	Hypertension(%Total)	Dyslipidaemia(%Total)	Previous CVD(%Total)	Previous Stroke(%Total)	Previous Heart failure(%Total)	Stage of CKD[Criteria]/ eGFR[Criteria] (ml/min/1.73 m^2^)	Outcomes (HR/RR/OR[95%CI])		Adjusting factors for all-cause mortality
							All-cause mortality	Cardiovascular events	
Masoudi et al. (26)	NR	NR	NR	NR	NR	CKD stage G1-5[a serum creatinine concentration ≥133 mol/L]	HR,0.86[0.75-0.98]	NR	markers of heart failure severity and comorbidities, differences in the treating physicians and hospitals, and the clustering of patients within hospitals
Roussel et al. (16)	NR	NR	NR	NR	NR	eGFR[MDRD] <30 30~44 45~59 30~59 <60	<30: HR,1.06[0.47-2.38] 30~44:HR,0.57[0.35-0.92] 45~59: HR,0.75[0.52-1.10] 30~59: HR,0.64[0.48-0.86] <60: HR,0.89[0.71-1.10]	NR	Multivariable adjustment and propensity score
Aguilar et al. (27)	NR	NR	NR	NR	NR	eGFR[MDRD] <60	HR,0.60[0.40-0.90]	NR	Propensity score adjusted model
Weir et al. (17)	NR	NR	NR	NR	40.4	eGFR[MDRD] <30 <45 <60	<30(Insulin): HR,0.31[0.10-0.91] <30(Glyburide): HR,0.21[0.07-0.67] <45(Insulin): HR,0.11[0.06-0.23] <45(Glyburide): HR,0.13[0.06-0.27] <60(Insulin): HR,0.13[0.08-0.20] <60(Glyburide) : HR,0.17[0.10-0.26]	NR	previous hypoglycaemic events, Charlson comorbidity index (1, 2 or ≥3), recent hospitalization, chronic liver disease, alcoholism,coronary artery disease, congestive heart failure, number of distinct medications used in the previous year (≤5, 6–10, 11–15, 16–20, 21–26 or ≥26), number of internist visits in the previous 5 years (≤5, 6–14 or≥15), concurrent use of corticosteroids, thiazide diuretics or atypical antipsychotics.
Ekström et al (28)	NR	51.1	21.4	NR	5.9	eGFR[MDRD] 30~44 45~59 ≥60	30~44:HR,1.02[0.84-1.24] 45~59: HR,0.87[0.77-0.99] ≥60: HR,0.87[0.81-0.94]	30~44:HR,1.0[0.83-1.19] 45~59: HR,0.94[0.84-1.05] ≥60: HR,0.98[0.92-1.05]	age, sex, diabetes duration, HbA1c, non-HDL-C, BMI, smoking, eGFR, multidose dispensation, previous hospitalisation, history of CVD and CHF, microalbuminuria, and treatment with antihypertensive agents, lipid-lowering agents and cardiac glycosides
Morgan et al. (29)	NR	NR	NR	NR	NR	CKD stage G1-5[a serum creatinine concentration >132 mol/L for males and >123 mol/L for females]	HR,0.61[0.53-0.69]	NR	Propensity score adjusted model
Fung et al. (30)	23.81	3.68	NR	NR	NR	CKD stage G1-3[eGFR] ≥90 60~89 30~59	HR,0.705[0.547-0.908]	HR,0.684[0.556-0.842]	Propensity score adjusted model
Hung et al. (15)	79.7	49.85	NR	25.95	24.85	CKD stage G 5[a serum creatinine concentration ≥530 mol/L]	HR,1.35[1.20-1.51]	NR	Age,sex, Diabetes duration, Comorbid disorders, Charlson comorbidity index score, Nephrologist visits, Antihypertensive drugs, Antidiabetic drugs, Geographic location, Propensity score
Marcum et al. (31)	77.1	47.91	NR	NR	6.29	CKD stage G 1-3[eGFR] ≥90 60~89 45~59 30~44	≥90:HR,0.59[0.50-0.70] 60~89:HR,0.65[0.60-0.71] 45~59:HR,0.80[0.71-0.91] 30~44:HR,0.72[0.51-1.01]	NR	Demographics,Health behavior,eGFR,Comorbid conditions,Laboratory results,Cardiovascular medications
Bergmark et al. (24)	85.66	76.7	NR	14.5	NR	Moderate/Severe CKD [eGFR] ≤45	HR,0.89[0.62-1.28]	NR	Propensity score adjusted model
Charytan et al. (25)	NR	NR	57.5	9.7	23.4	CKD stage G 1-5[eGFR] eGFR[CKD-EPI] ≥30 <30	CKD stage G1-3:HR,0.61[0.44-0.85] CKD stage G4-5:HR,0.83[0.54-1.24]	CKD stage G1-3:HR,0.70[0.53-0.90] CKD stage G4-5:HR,0.99[0.71-1.39]	Propensity score adjusted model
Kwon et al. (18)	49.0	NR	NR	NR	NR	eGFR[CKD-EPI] ≥45 30~44 <30	≥45:HR, 0.70[0.60-0.82] 30~44: HR, 0.64[0.47-0.86] <30: HR, 0.55[037-0.81]	NR	Propensity score adjusted model
Whitlock et al. (32)	61.9	34.9	33.2	NR	NR	CKD stage G 1-5[eGFR] eGFR[CKD-EPI] ≥90 60~89 45~59 30~44 <30	≥90: HR, 0.38[0.27-0.53] 60~89: HR, 0.42[0.31-0.56] 45~59: HR, 0.92[0.53-1.61] 30~44: HR, 0.85[0.46-1.57] <30: HR, 1.51[0.58-3.95]	≥90: HR, 0.64[0.52-0.86] 60~89: HR, 0.79[0.52-1.20] 45~59: HR, 0.86[0.45-1.64] 30~44: HR, 0.62[0.30-1.29] <30: HR, 0.56[0.18-1.69]	IPTW adjusted

RCT, randomized controlled trial; HbA1c, hemoglobin A1c; BMI, Body Mass Index; CKD, chronic kidney disease; CVD, cardiovascular disease; eGFR, estimated glomerular filtration rate; MDRD, Modification of Diet in Renal Disease; CKD-EPI, Chronic Kidney Disease Epidemiology Collaboration; OR, odds ratio; RR,risk ratio; HR, :Hazard Ratio; CI, confidence interval; IPTW, inverse probability of treatment weight; NR, not reported.

The 13 studies all had NOS scores ≥7 (mean= 8.8), which implied high quality (**Table S1**).

### Effect of Metformin on All-Cause Mortality in Patients With T2DM and CKD

#### Mild/Moderate CKD (eGFR≥ 30 ml/min/1.73m2)

**Figure 2:**
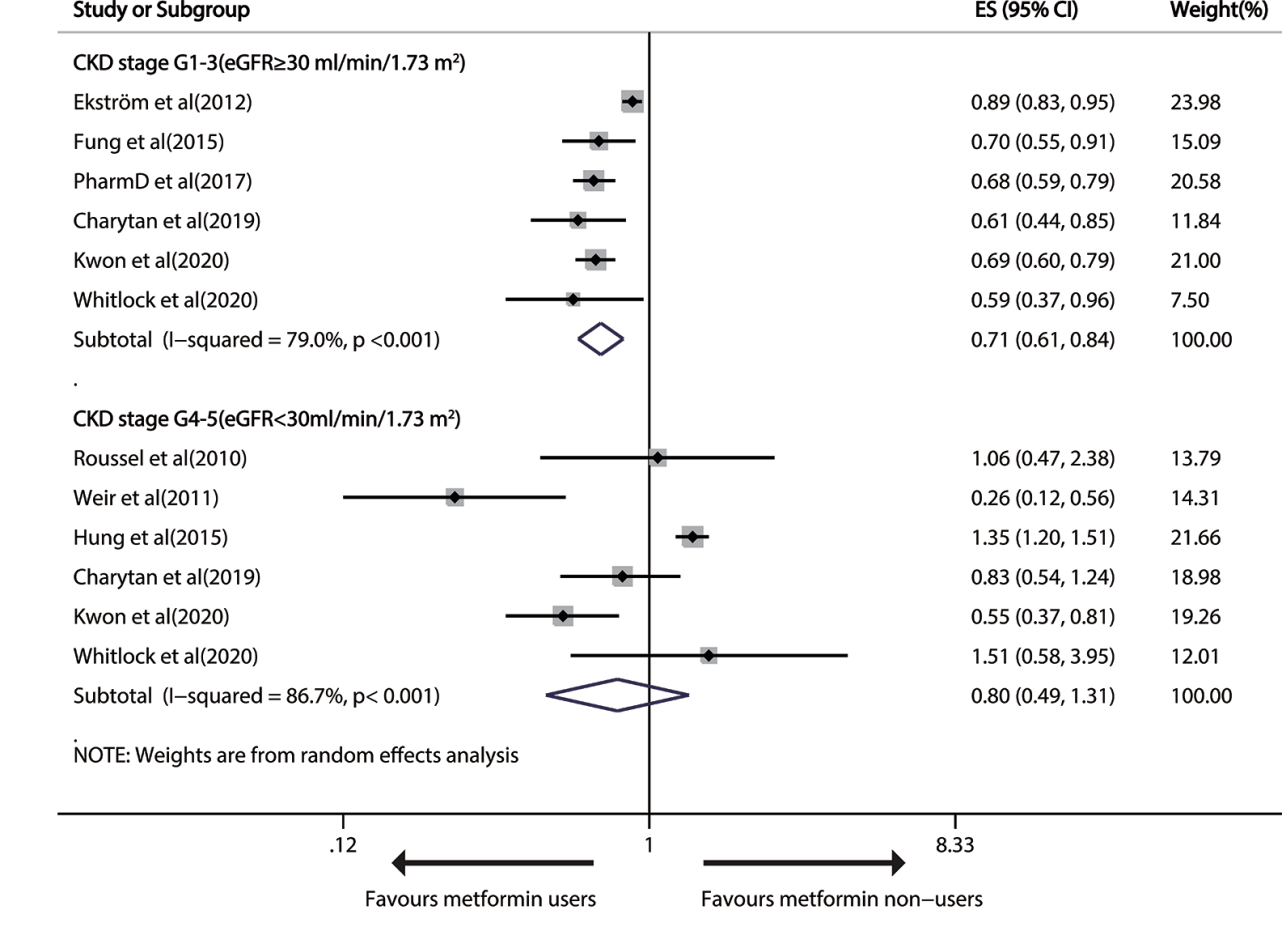
Forest plot of the risk of all-cause mortality in patients with type 1 diabetes mellitus (T2DM) and chronic kidney disease (CKD).

Six studies reported the effect of metformin use versus any other measure on all-cause mortality in patients with T2DM and mild/moderate CKD (18, 25, 28, 30–32). The pooled RR was 0.71 (95%CI, 0.61 to 0.84; P<0.001) in a random-effects model, with severe heterogeneity (I^2 =^ 79.0%; P<0.001; **Figure 2**).

Sensitivity analysis uncovered that heterogeneity did not disappear after the deletion of single studies. Significant publication bias was found by Egger’s test (P=0.043), but not by funnel plot inspection or Begg test (P=1) (**Figure S1**). During publication bias exploration based on the trim-and-fill approach, the probable missing data were not replaced, so the results were basically equal to a remarkably less risk of all-cause death after metformin treatment (RR 0.71, 95% CI 0.61–0.84; P<0.001). The GRADE determined a “low”-quality evidence that metformin prevented all-cause death in mild/modest CKD patients.

#### Advanced CKD (eGFR< 30 ml/min/1.73m^2^)

Six trails reported all-cause mortality in advanced CKD patients (15–18, 25, 32). Metformin use had no significant therapeutic effect on all-cause death (pooled RR: 0.80; 95%CI: 0.49, 1.31), with heavy between-study heterogeneity (I^2 =^ 86.7%; P<0.001; **Figure 2**). The effect was still insignificant in the stratified analysis by study design (**Figure S2**). The heterogeneity did not disappear after the exclusion of any single study.

The funnel plots showed no evident systematic bias between all-cause death and advanced CKD (Begg test, P= 0.707, Egger’s test, P=0.130; **Figure S3**).

#### Moderate CKD (30≤ eGFR< 59 ml/min/1.73m^2^)

**Figure 3:**
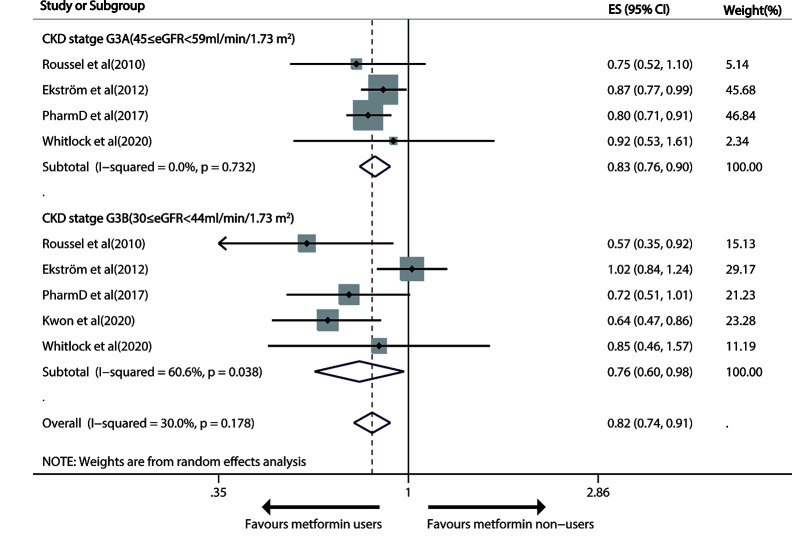
Forest plot of the risk of all-cause mortality in patients with type 1 diabetes mellitus (T2DM) and moderate chronic kidney disease (CKD) according to estimated glomerular filtration rate (eGFR).

Metformin use versus non-metformin measure led to a significant drop of 17% (pooled RR, 0.83; 95% CI, 0.76—0.90; P < 0.001) and 24% (0.76; 0.60—0.98; P=0.032) in all-cause death in patients with G3A CKD (45≤eGFR<59 ml/min/1.73m^2^) and G3B CKD (30≤ eGFR< 44 ml/min/1.73m^2^), respectively (**Figure 3**).

After one study (28) was deleted, the overall risk result slightly changed (HR 0.67, 95%CI: 0.55–0.82) in the random-effects model, with absolutely no heterogeneity (I^2 =^ 0, p=0.737). The funnel plots uncovered no severe systematic bias between all-cause death and G3B CKD (Begg test, P= 1, Egger’s test, P=0.219; **Figure S4**). GRADE also displayed a “low”-quality evidence that metformin avoided all-cause mortality in G3 CKD patients.

### Effect of Metformin Treatment Versus Non-Metformin Treatment on the Risk of Cardiovascular Events in Patients With T2DM and CKD

**Figure 4:**
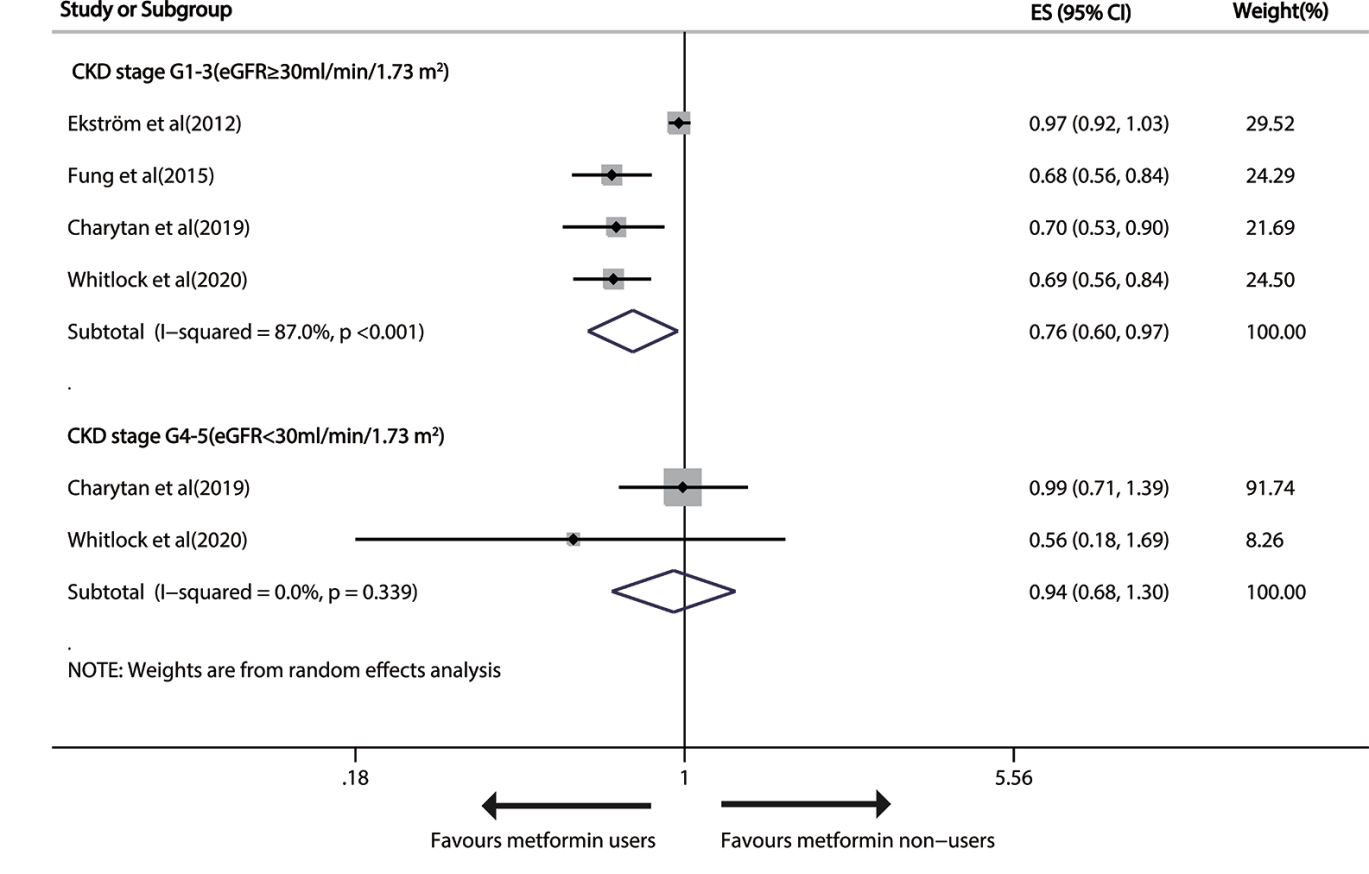
Forest plot of the risk of cardiovascular events in patients with type 1 diabetes mellitus (T2DM) and chronic kidney disease (CKD).

The 4 relevant studies (25, 28, 30, 32) on G1-3 CKD patients (eGFR≥30 ml/min/1.73m^2^) reported that metformin use induced a significant 24% decline (pooled RR, 0.76; 95% CI, 0.60—0.97; P=0.024) in susceptibility to cardiovascular events in a random-effects model with considerable heterogeneity (I^2 =^ 87.0%; P<0.001). But no significant effect of metformin use on advanced CKD was found (pooled RR: 0.94; 95%CI: 0.68, 1.30), without heterogeneity (**Figure 4**).

The evidence quality that metformin prevented cardiovascular events at stage G1-3 of CKD as determined with GRADE was “low”.

**Figure 5:**
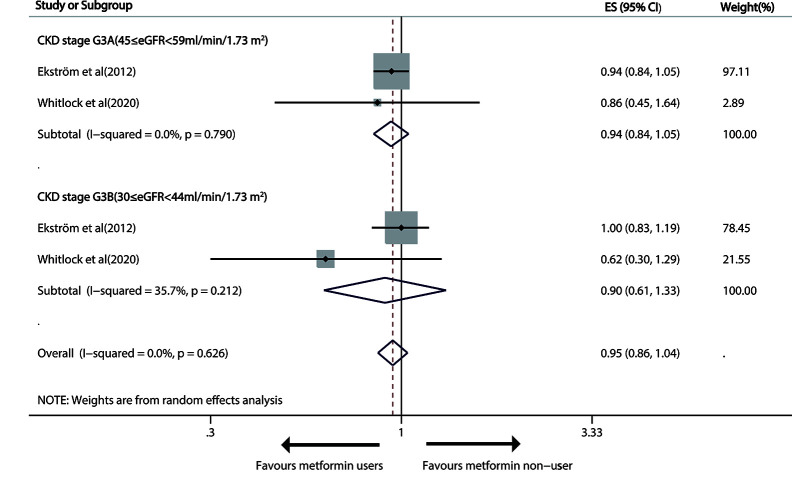
Forest plot of the risk of cardiovascular events in patients with type 1 diabetes mellitus (T2DM) and moderate chronic kidney disease (CKD) according to estimated glomerular filtration rate (eGFR). T2DM, type 1 diabetes mellitus; CKD, Chronic kidney disease; eGFR, estimated glomerular filtration rate.

As for moderate CKD (30≤eGFR<59 ml/min/1.73m^2^), metformin versus non-metformin treatment did not significantly reduce cardiovascular events in either G3A CKD (pooled RR: 0.94; 95%CI: 0.84, 1.05) or G3B CKD (pooled RR: 0.90; 95%CI: 0.61, 1.33; **Figure 5**).

## Discussion

All existing studies with 303,540 patients were searched, but evident positive relationship between metformin intake and all-cause death was found only at stage G1-3 of CKD. The risk reduction may be partially ascribed to the cardiovascular protective effects of metformin on coherent improvement in hypertension, dyslipidaemia and body mass index, despite the absence of HbA1c reduction found in one study (30). Metformin may decrease mortality following several biological mechanisms (33, 34): by lowering the insulin and insulin-like growth factor 1 signaling, mechanistically inhibiting rapamycin, weakening inflammation, reducing reactive oxygen species generation or DNA damage, or stimulating AMP-activated protein kinase. Metformin can experimentally initiate AMP-activated protein kinase and thus prevent diabetic lipoapoptosis of human coronary artery endothelial cells. Metformin can remove glycocalyx barrier in db/db mice, and relieve Ang-II-caused atheromatous plaque generation and aortic aneurysm in ApoE (−/−) mice. Metformin intake can remarkably weaken neointimal hyperplasia in fructose-caused insulin- resistant rats and myocardial reshaping and neutrophil recruitment after myocardial infarction in rats. Metformin also leads to a less risk of atrial fibrillations in T2DM patients probably by relieving myolysis and oxidative stress after atrial cell tachycardia.

Metformin versus non-metformin treatment resulted in 17% and 24% significant drop in all-cause death in CKD patients at stage G3A and G3B respectively. The risk reduction may not be ascribed to the cardiovascular protective effects of metformin use, which was seen in this review. A recent systematic review on health outcomes and conflicts related to metformin therapy summarized that metformin use can improve key clinical outcomes in T2DM patients with moderate CKD (10), which is similar to our result. This finding was partially based on the results from two OSs that presented death by CKD stage, which, however, was rated as possessing a modest risk of bias. According to Swedish National Diabetes Register, metformin-based measures resulted in a less risk of death in people with G3a CKD, but not with G3b CKD (HR 1.02, 95% CI 0.84–1.24) (28). Moreover, a cohort study enrolling American patients registered in Veterans Health Administration found that metformin use largely lowered the risk of all-cause mortality with the presence of eGFR at 45–59 mL/min/1.73 m^2^ (HR 0.80, 95%CI: 0.71–0.91), but not at 30–44 ml/min/1.73m^2^ (0.72, 0.51–1.01) (31). Whitlock et al. found metformin use was unrelated with smaller risk for all-cause death in CKD patients at stage of either G3A (HR 0.85, 95%CI: 0.46–1.57) or G3B (0.92, 0.53–1.61) (32). The deletion of one study (28) a bit changed the overall risk result in the random-effects model, and almost no heterogeneity was found. The differences can be ascribed to the discrepancies in study populations and comparator therapy.

No significant relationship was identified between metformin use and the end points of all-cause death or cardiovascular events in advanced CKD patients. The reasons why the mortality benefit from metformin versus any other measure is not considerable at stage of advanced CKD are unclear. One probable reason is that the cause of advanced CKD may be other than diabetic nephropathy and thus this comorbid condition may offer extra death risk factors that do not respond to OHA treatment, therefore restricting the death benefit of metformin seen in this population. Another possible reason is that the follow-up duration was too short to correctly uncover the actual chronic death benefits. A third potential reason might be that the number of patients with advanced CKD or ESKD on metformin is too small, and most studies involved less than 200 of patients with advanced CKD or ESKD on metformin except for one study (15). It is well-known that studies with smaller sample sizes might, very likely, lack sufficient statistical power to reveal the true association.

Heterogeneity of OSs cannot be avoided in any review and meta-analysis, owing to the differences in study design, patient characteristics and statistical approaches. Even strict criteria were used, and the enrolled articles stand for a comprehensive try to pool together published and unpublished studies of interest. Hence, single article effects were judged at the summary-level. Furthermore, the statistical approaches used in the OSs did not well solve the impact of unmeasured confounding elements on the overall effect estimation. Sensitivity analyses revealed the origins of heterogeneity were ascribed to the differences in study populations and comparator use.

This review has some merits, such as the large sample size, broad search scheme, and the accordance to Cochrane guidelines. Nevertheless, it also has some shortcomings. First, the absence of RCTs or unpublished articles may have biased our conclusions. Second, advancing patients who more likely to switch to glucose- reducing therapy during the study period were not excluded, which diluted the observations. Third, comparator uses were diverse and some new antidiabetic agents (e.g., sodium-glucose cotransporter-2 inhibitors, glucagon-like peptide 1 agonists) were basically not used by low-eGFR patients, so we cannot assess the impacts of metformin relative to these agents. Fourth, data were only about the dose and duration effect of metformin, case numbers, and person-years for dose–reaction meta-analyses. Fifth, we cannot well clarify the cause of CKD. As probably expected, the risks of these key clinical outcomes observed in diabetic CKD patients were largely different among the OSs, but we cannot conduct subgroup analysis because of the limited number of trails. Furthermore, Egger’s test for all-cause death in G1-3 CKD patients uncovered possible publication bias, which cannot be easily ascertained. In all, we may overestimate the real effect if insignificant articles were missed.

In conclusion, metformin use is related to significantly less risks of all-cause mortality and cardiovascular events in mild/modest CKD patients. However, RCTs with large sample sizes are warranted in the future to assess whether these key benefits extend to later stages of CKD by dose adjustment.

## Data Availability Statement

All datasets presented in this study are included in the article/**Supplementary Material**.

## Author Contributions

YH and ML performed the database research and statistical analysis. GK reviewed the conflicts in the databases obtained by YH and ML. YH wrote the first draft of the manuscript. XH, XP, LZ, and PF reviewed the manuscript critically for contents. All authors contributed to the article and approved the submitted version.

## Conflict of Interest

The authors declare that the research was conducted in the absence of any commercial or financial relationships that could be construed as a potential conflict of interest.

## Supplementary Material

Supplementary Figure 1 Funnel plot of the risk of all-cause mortality in patients with T2DM and Mild/moderate CKD.

Supplementary Figure 2 Forest plot of the risk of all-cause mortality in patients with T2DM and advanced CKD according to study design.

Supplementary Figure 3 Funnel plot of the risk of all-cause mortality in patients with T2DM and advanced CKD.

Supplementary Figure 4 Funnel plot of the risk of all-cause mortality in patients with T2DM and stage G3B CKD. T2DM, type 1 diabetes mellitus; CKD, Chronic kidney disease.

Supplementary Table 1 Quality assessment of observational studies included in the meta-analysis by NOS. NOS, Newcastle–Ottawa scale.
