# Supplementary material for: Metformin Use and Risk of All-Cause Mortality and Cardiovascular Events in Patients With Chronic Kidney Disease—A Systematic Review and Meta-Analysis
Source: Front Endocrinol (Lausanne). 2020 Oct 7;11:559446. doi: 10.3389/fendo.2020.559446 (PMC7575818; doi:10.3389/fendo.2020.559446)
Supplement: Supplementary file 1 [file DataSheet_1.docx]

**Search strategy**

**Pubmed**

#1 NIDDM[Title/Abstract]
#2 MODY[Title/Abstract]
#3 Diabetes mellitus, type 2[MeSH Terms]

#4 glucose intolerance[MeSH Terms]
#5 “late onset diabet*”[Title/Abstract]
#6 “maturity onset diabet*”[Title/Abstract]
#7 “non insulin* depend*”[Title/Abstract] OR “noninsulin* depend*”[Title/Abstract]

#8 “type 2 diabet*”[Title/Abstract]
#9 “type II diabet*”[Title/Abstract]
#10 “insulin resistance”[MeSH Terms]

#11 “insulin resistance”[Title/Abstract]
#12 T2D[Title/Abstract] OR T2DM[Title/Abstract]
#13 #1 OR #29 OR #3 OR #4 OR #5 OR #6 OR #7 OR #8 OR #9 OR #10 OR #11 OR #12

#[14](http://www.ncbi.nlm.nih.gov/pubmed/advanced) uremia[Title/Abstract]

#[15](http://www.ncbi.nlm.nih.gov/pubmed/advanced) [uraemia](http://www.ncbi.nlm.nih.gov/pubmed/advanced)[Title/Abstract]

#[16](http://www.ncbi.nlm.nih.gov/pubmed/advanced) “kidney* failure*”[Title/Abstract]

#17 “renal failure*”[Title/Abstract]

#18 “chronic kidney”[Title/Abstract] OR “chronic renal”[Title/Abstract]

#19 CKD [Title/Abstract] OR CKF[Title/Abstract] OR CRD [Title/Abstract] OR CRF [Title/Abstract] OR ESKD [Title/Abstract] OR ESRD [Title/Abstract] OR ESKF[Title/Abstract] OR ESRF [Title/Abstract]

#[20](http://www.ncbi.nlm.nih.gov/pubmed/advanced) “endstage kidney”[Title/Abstract] OR “endstage renal”[Title/Abstract] OR “end-stage kidney”[Title/Abstract] OR “end-stage renal”[Title/Abstract]

#[21](http://www.ncbi.nlm.nih.gov/pubmed/advanced) Kidney Failure, Chronic[MeSH Terms]

#22 #14 OR #15 OR #16 OR #17 OR #18 OR #19 OR #20 OR #21

#23 “renal replacement therapy”[Title/Abstract]

#24 “artificial kidney”[Title/Abstract]

#25 ultrafiltration[Title/Abstract]

#26 dialysis[Title/Abstract]

#27 ultrafiltrat*[Title/Abstract] OR dialy*[Title/Abstract]

#28 “kidney* replac*”[Title/Abstract]

#29 “kidney* artificial*”[Title/Abstract]

#30 “peritoneal dialysis”[Title/Abstract] OR CAPD [Title/Abstract] OR CCPD [Title/Abstract] OR APD [Title/Abstract]

#31 haemodialysis [Title/Abstract] OR hemodialysis [Title/Abstract]

#32 haemofiltration [Title/Abstract] OR hemofiltration [Title/Abstract]

#33 haemodiafiltration [Title/Abstract] OR hemodiafiltration [Title/Abstract]

#34 Renal Dialysis[MeSH Terms]

#35 Hemofiltration[MeSH Terms]

#36 peritoneal Dialysis[MeSH Terms]

#37 HD[Title/Abstract] OR HP[Title/Abstract] OR HF[Title/Abstract] OR HDF[Title/Abstract] OR CVVH[Title/Abstract] OR CAVH[Title/Abstract] OR CVVHD[Title/Abstract] OR CVVHDF[Title/Abstract]

[#38](http://www.ncbi.nlm.nih.gov/pubmed/advanced) [#](http://www.ncbi.nlm.nih.gov/pubmed/advanced)23 OR [#24](http://www.ncbi.nlm.nih.gov/pubmed/advanced) OR [#25](http://www.ncbi.nlm.nih.gov/pubmed/advanced) OR [#26](http://www.ncbi.nlm.nih.gov/pubmed/advanced) OR [#27](http://www.ncbi.nlm.nih.gov/pubmed/advanced) OR [#28](http://www.ncbi.nlm.nih.gov/pubmed/advanced) OR [#29](http://www.ncbi.nlm.nih.gov/pubmed/advanced) OR [#30](http://www.ncbi.nlm.nih.gov/pubmed/advanced) OR [#31](http://www.ncbi.nlm.nih.gov/pubmed/advanced) OR [#32](http://www.ncbi.nlm.nih.gov/pubmed/advanced) OR [#33](http://www.ncbi.nlm.nih.gov/pubmed/advanced) OR [#34](http://www.ncbi.nlm.nih.gov/pubmed/advanced) OR [#35](http://www.ncbi.nlm.nih.gov/pubmed/advanced) OR [#36](http://www.ncbi.nlm.nih.gov/pubmed/advanced) OR [#37](http://www.ncbi.nlm.nih.gov/pubmed/advanced)

[#39](http://www.ncbi.nlm.nih.gov/pubmed/advanced) #22 OR #38

[#40](http://www.ncbi.nlm.nih.gov/pubmed/advanced) Metformin[Title/Abstract]

[#41](http://www.ncbi.nlm.nih.gov/pubmed/advanced) Glucophage[Title/Abstract]

[#42](http://www.ncbi.nlm.nih.gov/pubmed/advanced) dimethylbiguanidine[Title/Abstract]

[#43](http://www.ncbi.nlm.nih.gov/pubmed/advanced) dimethylguanylguanidine[Title/Abstract]

[#44](http://www.ncbi.nlm.nih.gov/pubmed/advanced) dimethylbiguanidium[Title/Abstract]

[#45](http://www.ncbi.nlm.nih.gov/pubmed/advanced) glucovance[Title/Abstract]

[#46](http://www.ncbi.nlm.nih.gov/pubmed/advanced) Metformin[MeSH Terms]

[#47](http://www.ncbi.nlm.nih.gov/pubmed/advanced) [#40](http://www.ncbi.nlm.nih.gov/pubmed/advanced) OR [#41](http://www.ncbi.nlm.nih.gov/pubmed/advanced) OR [#42](http://www.ncbi.nlm.nih.gov/pubmed/advanced) OR [#43](http://www.ncbi.nlm.nih.gov/pubmed/advanced) OR [#44](http://www.ncbi.nlm.nih.gov/pubmed/advanced) OR [#45](http://www.ncbi.nlm.nih.gov/pubmed/advanced) OR [#46](http://www.ncbi.nlm.nih.gov/pubmed/advanced)

#48 “death”[Title/Abstract]

#49 “decease”[Title/Abstract]

#50 “demise”[Title/Abstract]

#51 “die”[Title/Abstract]

#52 “dead”[Title/Abstract]

#53 “loss of life”[Title/Abstract]

#54 “life time”[Title/Abstract]

#55 “lifetime”[Title/Abstract]

#56 “life span”[Title/Abstract]

#57 “duration of life”[Title/Abstract]

#58 “mortality”[Title/Abstract]

#59 “mortalit*”[Title/Abstract]

#60 “length of life”[Title/Abstract]

#61 “life duration” [Title/Abstract]

#62 “longevity”[Title/Abstract]

#63 “natural life”[Title/Abstract]

#64 “operating life”[Title/Abstract]

#65 “survival”[Title/Abstract]

#66 “death”[MeSH Terms] OR “mortality”[MeSH Terms]

#67 #48 OR #49 OR #50 OR #51 OR #52 OR #53 OR #54 OR #55 OR #56 OR #57 OR #58 OR #59 OR #60 OR #61 OR #62 OR #63 OR #64 OR #65 OR #66

#68 (animal[mh] not human[mh])

#69 comment[Publication Type] OR editorial[Publication Type] OR letter[Publication Type]

#70 #13 AND #39 AND #47 AND #67 NOT#68 NOT #69

**Embase**

#1 NIDDM:ab,ti
#2 MODY:ab,ti
#3 ‘non insulin dependent diabetes mellitus’/exp

#4 ‘glucose intolerance’/exp
#5 ‘late onset diabet$’:ab,ti
#6 ‘maturity onset diabet$’:ab,ti
#7 ‘non insulin$ depend$’:ab,ti OR ‘noninsulin$ depend$’:ab,ti

#8 ‘type 2 diabet$’:ab,ti
#9 ‘type II diabet$’:ab,ti
#10 ‘insulin resistance’/exp

#11 ‘insulin resistance’:ab,ti
#12 T2D:ab,ti OR T2DM:ab,ti
#13 #1 OR #29 OR #3 OR #4 OR #5 OR #6 OR #7 OR #8 OR #9 OR #10 OR #11 OR #12

#[14](http://www.ncbi.nlm.nih.gov/pubmed/advanced) uremia:ab,ti

#[15](http://www.ncbi.nlm.nih.gov/pubmed/advanced) [uraemia](http://www.ncbi.nlm.nih.gov/pubmed/advanced):ab,ti

#[16](http://www.ncbi.nlm.nih.gov/pubmed/advanced) ‘kidney$ failure$’:ab,ti

#17 ‘renal failure$’:ab,ti

#18 ‘chronic kidney’:ab,ti OR ‘chronic renal’:ab,ti

#19 CKD:ab,ti OR CKF:ab,ti OR CRD:ab,ti OR CRF:ab,ti OR ESKD:ab,ti OR ESRD:ab,ti OR ESKF:ab,ti OR ESRF:ab,ti

#[20](http://www.ncbi.nlm.nih.gov/pubmed/advanced) ‘endstage kidney’:ab,ti OR ‘endstage renal’:ab,ti OR ‘end-stage kidney’:ab,ti OR ‘end-stage renal’:ab,ti

#[21](http://www.ncbi.nlm.nih.gov/pubmed/advanced) ‘chronic kidney failure’/exp

#22 #14 OR #15 OR #16 OR #17 OR #18 OR #19 OR #20 OR #21

#23 ‘renal replacement therapy’:ab,ti

#24 ‘artificial kidney’:ab,ti

#25 ultrafiltration:ab,ti

#26 dialysis:ab,ti

#27 ultrafiltrat$:ab,ti OR dialy$:ab,ti

#28 ‘kidney$ replac$’:ab,ti

#29 ‘kidney$ artificial$’:ab,ti

#30 ‘peritoneal dialysis’:ab,ti OR CAPD:ab,ti OR CCPD:ab,ti OR APD:ab,ti

#31 haemodialysis:ab,ti OR hemodialysis:ab,ti

#32 haemofiltration:ab,ti OR hemofiltration:ab,ti

#33 haemodiafiltration:ab,ti OR hemodiafiltration:ab,ti

#34 ‘renal replacement therapy’/exp

#35 ‘Hemofiltration’/exp

#36 ‘peritoneal Dialysis’/exp OR ‘hemodialysis’/exp

#37 HD:ab,ti OR HP:ab,ti OR HF:ab,ti OR HDF:ab,ti OR CVVH:ab,ti OR CAVH:ab,ti OR CVVHD:ab,ti OR CVVHDF:ab,ti

[#38](http://www.ncbi.nlm.nih.gov/pubmed/advanced) [#](http://www.ncbi.nlm.nih.gov/pubmed/advanced)23 OR [#24](http://www.ncbi.nlm.nih.gov/pubmed/advanced) OR [#25](http://www.ncbi.nlm.nih.gov/pubmed/advanced) OR [#26](http://www.ncbi.nlm.nih.gov/pubmed/advanced) OR [#27](http://www.ncbi.nlm.nih.gov/pubmed/advanced) OR [#28](http://www.ncbi.nlm.nih.gov/pubmed/advanced) OR [#29](http://www.ncbi.nlm.nih.gov/pubmed/advanced) OR [#30](http://www.ncbi.nlm.nih.gov/pubmed/advanced) OR [#31](http://www.ncbi.nlm.nih.gov/pubmed/advanced) OR [#32](http://www.ncbi.nlm.nih.gov/pubmed/advanced) OR [#33](http://www.ncbi.nlm.nih.gov/pubmed/advanced) OR [#34](http://www.ncbi.nlm.nih.gov/pubmed/advanced) OR [#35](http://www.ncbi.nlm.nih.gov/pubmed/advanced) OR [#36](http://www.ncbi.nlm.nih.gov/pubmed/advanced) OR [#37](http://www.ncbi.nlm.nih.gov/pubmed/advanced)

[#39](http://www.ncbi.nlm.nih.gov/pubmed/advanced) #22 OR #38

[#40](http://www.ncbi.nlm.nih.gov/pubmed/advanced) Metformin:ab,ti

[#41](http://www.ncbi.nlm.nih.gov/pubmed/advanced) Glucophage:ab,ti

[#42](http://www.ncbi.nlm.nih.gov/pubmed/advanced) dimethylbiguanidine:ab,ti

[#43](http://www.ncbi.nlm.nih.gov/pubmed/advanced) dimethylguanylguanidine:ab,ti

[#44](http://www.ncbi.nlm.nih.gov/pubmed/advanced) dimethylbiguanidium:ab,ti

[#45](http://www.ncbi.nlm.nih.gov/pubmed/advanced) glucovance:ab,ti

[#46](http://www.ncbi.nlm.nih.gov/pubmed/advanced) ‘Metformin’/exp

[#47](http://www.ncbi.nlm.nih.gov/pubmed/advanced) [#40](http://www.ncbi.nlm.nih.gov/pubmed/advanced) OR [#41](http://www.ncbi.nlm.nih.gov/pubmed/advanced) OR [#42](http://www.ncbi.nlm.nih.gov/pubmed/advanced) OR [#43](http://www.ncbi.nlm.nih.gov/pubmed/advanced) OR [#44](http://www.ncbi.nlm.nih.gov/pubmed/advanced) OR [#45](http://www.ncbi.nlm.nih.gov/pubmed/advanced) OR [#46](http://www.ncbi.nlm.nih.gov/pubmed/advanced)

#48 ‘death’:ab,ti

#49 ‘decease’:ab,ti

#50 ‘demise’:ab,ti

#51 ‘die’:ab,ti

#52 ‘dead’:ab,ti

#53 ‘loss of life’:ab,ti

#54 ‘life time’:ab,ti

#55 ‘lifetime’:ab,ti

#56 ‘life span’:ab,ti

#57 ‘duration of life’:ab,ti

#58 ‘mortality’:ab,ti

#59 ‘mortalit$’:ab,ti

#60 ‘length of life’:ab,ti

#61 ‘life duration’:ab,ti

#62 ‘longevity’:ab,ti

#63 ‘natural life’:ab,ti

#64 ‘operating life’:ab,ti

#65 ‘survival’:ab,ti

#66 ‘death’/exp

#67 ‘mortality’/exp

#68 #48 OR #49 OR #50 OR #51 OR #52 OR #53 OR #54 OR #55 OR #56 OR #57 OR #58 OR #59 OR #60 OR #61 OR #62 OR #63 OR #64 OR #65 OR #66 OR #67

#68 #13 AND #39 AND #47 AND #68

Lim #68 to human

**Cochrane**

#1 NIDDM:ti,ab,kw
#2 MODY:ti,ab,kw
#3 MeSH descriptor:[Diabetes mellitus, type 2] explode all trees

#4 MeSH descriptor:[glucose intolerance] explode all trees
#5 “late onset diabet*”:ti,ab,kw
#6 “maturity onset diabet*”:ti,ab,kw
#7 “non insulin* depend*”:ti,ab,kw OR “noninsulin* depend*”:ti,ab,kw

#8 “type 2 diabet*”:ti,ab,kw
#9 “type II diabet*”:ti,ab,kw
#10 MeSH descriptor:[ insulin resistance] explode all trees

#11 “insulin resistance”:ti,ab,kw
#12 T2D:ti,ab,kw OR T2DM:ti,ab,kw
#13 #1 OR #29 OR #3 OR #4 OR #5 OR #6 OR #7 OR #8 OR #9 OR #10 OR #11 OR #12

#[14](http://www.ncbi.nlm.nih.gov/pubmed/advanced) uremia:ti,ab,kw

#[15](http://www.ncbi.nlm.nih.gov/pubmed/advanced) [uraemia](http://www.ncbi.nlm.nih.gov/pubmed/advanced):ti,ab,kw

#[16](http://www.ncbi.nlm.nih.gov/pubmed/advanced) “kidney* failure*”:ti,ab,kw

#17 “renal failure*”:ti,ab,kw

#18 “chronic kidney”:ti,ab,kw OR “chronic renal”:ti,ab,kw

#19 CKD:ti,ab,kw OR CKF:ti,ab,kw OR CRD:ti,ab,kw OR CRF:ti,ab,kw OR ESKD:ti,ab,kw OR ESRD:ti,ab,kw OR ESKF:ti,ab,kw OR ESRF:ti,ab,kw

#[20](http://www.ncbi.nlm.nih.gov/pubmed/advanced) “endstage kidney”:ti,ab,kw OR “endstage renal”:ti,ab,kw OR “end-stage kidney”:ti,ab,kw OR “end-stage renal”:ti,ab,kw

#[21](http://www.ncbi.nlm.nih.gov/pubmed/advanced) MeSH descriptor:[Kidney Failure, Chronic] explode all trees

#22 #14 OR #15 OR #16 OR #17 OR #18 OR #19 OR #20 OR #21

#23 “renal replacement therapy”:ti,ab,kw

#24 “artificial kidney”:ti,ab,kw

#25 ultrafiltration:ti,ab,kw

#26 dialysis:ti,ab,kw

#27 ultrafiltrat*:ti,ab,kw OR dialy*:ti,ab,kw

#28 “kidney* replac*”:ti,ab,kw

#29 “kidney* artificial*”:ti,ab,kw

#30 “peritoneal dialysis”:ti,ab,kw OR CAPD:ti,ab,kw OR CCPD:ti,ab,kw OR APD:ti,ab,kw

#31 haemodialysis:ti,ab,kw OR hemodialysis:ti,ab,kw

#32 haemofiltration:ti,ab,kw OR hemofiltration:ti,ab,kw

#33 haemodiafiltration:ti,ab,kw OR hemodiafiltration:ti,ab,kw

#34 MeSH descriptor:[Renal Dialysis] explode all trees

#35 MeSH descriptor:[Hemofiltration] explode all trees

#36 MeSH descriptor:[peritoneal Dialysis] explode all trees

#37 HD:ti,ab,kw OR HP:ti,ab,kw OR HF:ti,ab,kw OR HDF:ti,ab,kw OR CVVH:ti,ab,kw OR CAVH:ti,ab,kw OR CVVHD:ti,ab,kw OR CVVHDF:ti,ab,kw

[#38](http://www.ncbi.nlm.nih.gov/pubmed/advanced) [#](http://www.ncbi.nlm.nih.gov/pubmed/advanced)23 OR [#24](http://www.ncbi.nlm.nih.gov/pubmed/advanced) OR [#25](http://www.ncbi.nlm.nih.gov/pubmed/advanced) OR [#26](http://www.ncbi.nlm.nih.gov/pubmed/advanced) OR [#27](http://www.ncbi.nlm.nih.gov/pubmed/advanced) OR [#28](http://www.ncbi.nlm.nih.gov/pubmed/advanced) OR [#29](http://www.ncbi.nlm.nih.gov/pubmed/advanced) OR [#30](http://www.ncbi.nlm.nih.gov/pubmed/advanced) OR [#31](http://www.ncbi.nlm.nih.gov/pubmed/advanced) OR [#32](http://www.ncbi.nlm.nih.gov/pubmed/advanced) OR [#33](http://www.ncbi.nlm.nih.gov/pubmed/advanced) OR [#34](http://www.ncbi.nlm.nih.gov/pubmed/advanced) OR [#35](http://www.ncbi.nlm.nih.gov/pubmed/advanced) OR [#36](http://www.ncbi.nlm.nih.gov/pubmed/advanced) OR [#37](http://www.ncbi.nlm.nih.gov/pubmed/advanced)

[#39](http://www.ncbi.nlm.nih.gov/pubmed/advanced) #22 OR #38

[#40](http://www.ncbi.nlm.nih.gov/pubmed/advanced) Metformin:ti,ab,kw

[#41](http://www.ncbi.nlm.nih.gov/pubmed/advanced) Glucophage:ti,ab,kw

[#42](http://www.ncbi.nlm.nih.gov/pubmed/advanced) dimethylbiguanidine:ti,ab,kw

[#43](http://www.ncbi.nlm.nih.gov/pubmed/advanced) dimethylguanylguanidine:ti,ab,kw

[#44](http://www.ncbi.nlm.nih.gov/pubmed/advanced) dimethylbiguanidium:ti,ab,kw

[#45](http://www.ncbi.nlm.nih.gov/pubmed/advanced) glucovance:ti,ab,kw

[#46](http://www.ncbi.nlm.nih.gov/pubmed/advanced) MeSH descriptor:[Metformin] explode all trees

[#47](http://www.ncbi.nlm.nih.gov/pubmed/advanced) [#40](http://www.ncbi.nlm.nih.gov/pubmed/advanced) OR [#41](http://www.ncbi.nlm.nih.gov/pubmed/advanced) OR [#42](http://www.ncbi.nlm.nih.gov/pubmed/advanced) OR [#43](http://www.ncbi.nlm.nih.gov/pubmed/advanced) OR [#44](http://www.ncbi.nlm.nih.gov/pubmed/advanced) OR [#45](http://www.ncbi.nlm.nih.gov/pubmed/advanced) OR [#46](http://www.ncbi.nlm.nih.gov/pubmed/advanced)

#48 “death”:ti,ab,kw

#49 “decease”:ti,ab,kw

#50 “demise”:ti,ab,kw

#51 “die”:ti,ab,kw

#52 “dead”:ti,ab,kw

#53 “loss of life”:ti,ab,kw

#54 “life time”:ti,ab,kw

#55 “lifetime”:ti,ab,kw

#56 “life span”:ti,ab,kw

#57 “duration of life”:ti,ab,kw

#58 “mortality”:ti,ab,kw

#59 “mortalit*”:ti,ab,kw

#60 “length of life”:ti,ab,kw

#61 “life duration” :ti,ab,kw

#62 “longevity”:ti,ab,kw

#63 “natural life”:ti,ab,kw

#64 “operating life”:ti,ab,kw

#65 “survival”:ti,ab,kw

#66 MeSH descriptor:[death] explode all trees

#67 #48 OR #49 OR #50 OR #51 OR #52 OR #53 OR #54 OR #55 OR #56 OR #57 OR #58 OR #59 OR #60 OR #61 OR #62 OR #63 OR #64 OR #65 OR #66

#68 #13 AND #39 AND #47 AND #67

#69 Limit #68 to clinical trails

**Web of Science**

**#1** TS=(NIDDM OR MODY OR “glucose intolerance” OR “late onset diabet*” OR “maturity onset diabet*” OR “non insulin* depend*” OR “noninsulin* depend*” OR “type 2 diabet*” OR “type II diabet*” OR “insulin resistance” OR T2D OR T2DM)

**#2** TS=(uremia OR [uraemia](http://www.ncbi.nlm.nih.gov/pubmed/advanced) OR “kidney* failure*” OR “renal failure*” OR “chronic kidney” OR “chronic renal” OR CKD OR CKF OR CRD OR CRF OR ESKD OR ESRD OR ESKF OR ESRF OR “endstage kidney” OR “endstage renal” OR “end-stage kidney” OR “end-stage renal”)

**#3** TS=(“renal replacement therapy” OR “artificial kidney” OR ultrafiltration OR dialysis OR ultrafiltrat* OR dialy* OR “kidney* replac*” OR “kidney* artificial*” OR “peritoneal dialysis” OR CAPD OR CCPD OR APD OR haemodialysis OR hemodialysis OR haemofiltration OR hemofiltration OR haemodiafiltration OR hemodiafiltration OR HD OR HP OR HF OR HDF OR CVVH OR CAVH OR CVVHD OR CVVHDF)

#4 #2 OR #3

#5 TS=(Metformin OR Glucophage OR dimethylbiguanidine OR dimethylguanylguanidine OR dimethylbiguanidium OR glucovance)

#6 TS=( ‘death’ OR ‘decease’ OR ‘demise’ OR ‘die’ OR ‘dead’ OR ‘loss of life’ OR ‘life time’ OR ‘lifetime’ OR ‘life span’ OR ‘duration of life’ OR ‘mortality’ OR ‘mortalit$’ OR ‘length of life’ OR ‘life duration’ OR ‘longevity’ OR ‘natural life’ OR ‘operating life’ OR ‘survival’)

#7 #1 and #4 and #5 and #6

Timespan:All years.

Search language=Auto DocType=All document types; Language=All languages
