# Supplementary material for: Metformin Use and Risk of All-Cause Mortality and Cardiovascular Events in Patients With Chronic Kidney Disease—A Systematic Review and Meta-Analysis
Source: Front Endocrinol (Lausanne). 2020 Oct 7;11:559446. doi: 10.3389/fendo.2020.559446 (PMC7575818; doi:10.3389/fendo.2020.559446)
Supplement: Supplementary Table 1 — Quality assessment of observational studies included in the meta-analysis by NOS. NOS, Newcastle–Ottawa scale. [file Table_1.doc]

Table S1 Quality assessment of studies using Newcastle-Ottawa Scale for Cohort Studies for all-cause mortality

| NOS scale | Masoudi et al (2005) | Aguilar et al  (2011) | Ekström et al  (2012) | Morgan et al (2014) | Fung et al  (2015) | Hung et al  (2015) | PharmD et al(2017) | Kwon et al  (2020) | Whitlock et al (2020) |
| --- | --- | --- | --- | --- | --- | --- | --- | --- | --- |
| A-Selection (maximum 4*) |  |  |  |  |  |  |  |  |  |
| 1.Representativeness of general  community population | * | * | * | * | * | * | * | * | * |
| 2.The reference group was drawn  from the same community | * | * | * | * | * | * | * | * | * |
| 3.Ascertainment the exposure of metformin therapy | * | * | * | * | * | * | * | * | * |
| 4.Outcome of interest was not present at baseline | * | * | * | * | * | * | * | * | * |
| B-Comparability (maximum 2*) |  |  |  |  |  |  |  |  |  |
| 5.Controlled for age | 0 | * | * | * | * | * | * | * | 0 |
| 6.Controlled for 2 or more variables | * | * | * | * | * | * | * | * | * |
| C-Outcome (maximum 3*) |  |  |  |  |  |  |  |  |  |
| 7. Outcome of interest was certificated by hospital or local municipal registration | * | * | * | * | * | * | * | * | * |
| 8. Adequate duration of follow-up(one year) | * | * | * | * | * | * | * | * | * |
| 9.Adequacy of follow-up rate (>90%) of cohorts | * | * | * | * | * | * | * | * | * |
| Total scores (maximum 9*) | 8 | 9 | 9 | 9 | 9 | 9 | 9 | 9 | 8 |

“*” meant the study was corresponded to the NOS criteria,” 0” meant the study wasn’t correspond to the NOS criteria

Table S1 Quality assessment of studies using Newcastle-Ottawa Scale for subanalyses or post-hoc analyses of RCTs for all-cause mortality

| NOS scale | Roussel et al  (2010) | Bergmark et al(2019) | Charytan et al(2019) |  |  |  |  |  |  |
| --- | --- | --- | --- | --- | --- | --- | --- | --- | --- |
| A-Selection (maximum 4*) |  |  |  |  |  |  |  |  |  |
| 1.Representativeness of general  community population | * | * | * |  |  |  |  |  |  |
| 2.The reference group was drawn  from the same community | * | * | * |  |  |  |  |  |  |
| 3.Ascertainment the exposure of metformin therapy | * | * | * |  |  |  |  |  |  |
| 4.Outcome of interest was not present at baseline | * | * | * |  |  |  |  |  |  |
| B-Comparability (maximum 2*) |  |  |  |  |  |  |  |  |  |
| 5.Controlled for age | * | * | * |  |  |  |  |  |  |
| 6.Controlled for 2 or more variables | * | * | * |  |  |  |  |  |  |
| C-Outcome (maximum 3*) |  |  |  |  |  |  |  |  |  |
| 7. Outcome of interest was certificated by hospital or local municipal registration | * | * | * |  |  |  |  |  |  |
| 8. Adequate duration of follow-up(one year) | * | * | * |  |  |  |  |  |  |
| 9.Adequacy of follow-up rate (>90%) of cohorts | * | * | * |  |  |  |  |  |  |
| Total scores (maximum 9*) | 9 | 9 | 9 |  |  |  |  |  |  |

“*” meant the study was corresponded to the NOS criteria,” 0” meant the study wasn’t correspond to the NOS criteria

Table S1 Quality assessment of studies using Newcastle-Ottawa Scale for Case-Control study for all-cause mortality

| NOS scale | Weir et al  (2011) |  |  |  |  |  |  |  |  |
| --- | --- | --- | --- | --- | --- | --- | --- | --- | --- |
| A-Selection (maximum 4*) |  |  |  |  |  |  |  |  |  |
| 1.The case definition was adequate | * |  |  |  |  |  |  |  |  |
| 2.The cases were consecutive or are obviously representative series of cases | * |  |  |  |  |  |  |  |  |
| 3.The reference group was drawn from the same community | * |  |  |  |  |  |  |  |  |
| 4. No history of endpoints were present in the reference group | * |  |  |  |  |  |  |  |  |
| B-Comparability (maximum 2*) |  |  |  |  |  |  |  |  |  |
| 5.Controlled for age | 0 |  |  |  |  |  |  |  |  |
| 6.Controlled for 2 or more variables | * |  |  |  |  |  |  |  |  |
| C-Exposure (maximum 3*) |  |  |  |  |  |  |  |  |  |
| 7. Exposure was certificated by hospital or local municipal registration | * |  |  |  |  |  |  |  |  |
| 8. Same method of ascertainment for cases and controls | * |  |  |  |  |  |  |  |  |
| 9. Same non-response rate for both groups | * |  |  |  |  |  |  |  |  |
| Total scores (maximum 9*) | 8 |  |  |  |  |  |  |  |  |

“*” meant the study was corresponded to the NOS criteria,” 0” meant the study wasn’t correspond to the NOS criteria
